# Head circumference growth in children with Autism Spectrum Disorder: trend and clinical correlates in the first five years of life

**DOI:** 10.3389/fpsyt.2024.1431693

**Published:** 2024-08-06

**Authors:** Lara Cirnigliaro, Luisa Clericò, Lorenza Chiara Russo, Adriana Prato, Manuela Caruso, Renata Rizzo, Rita Barone

**Affiliations:** ^1^ Child Neurology and Psychiatry Unit, Department of Clinical and Experimental Medicine, University of Catania, Catania, Italy; ^2^ Pediatric Endocrinology and Diabetology Center, Department of Clinical and Experimental Medicine, University of Catania, Catania, Italy; ^3^ Research Unit of Rare Diseases and Neurodevelopmental Disorders, Oasi Research Institute-IRCCS, Troina, Italy

**Keywords:** Autism Spectrum Disorder, macrocephaly, head circumference, head growth trajectory, neurodevelopment, endophenotype

## Abstract

**Background:**

Macrocephaly is described in almost 15% of children with Autism Spectrum Disorder (ASD). Relationships between head growth trajectories and clinical findings in ASD children show a high degree of variability, highlighting the complex heterogeneity of the disorder.

**Objectives:**

The aim of this study was to measure differences of the early growth trajectory of head circumference (HC) in children with ASD and macrocephaly compared to ASD normocephalic children, examining clinical correlates in the two groups of patients.

**Methods:**

HC data were collected from birth to 5 years of age in a sample of children with a confirmed diagnosis of ASD. Participants were classified into two groups: ASD macrocephaly (ASD-M, Z-scores ≥1.88 in at least two consecutive HC measurements), and ASD non-macrocephaly (ASD-N). Based on the distribution of HC measurements (Z-scores), five age groups were identified for the longitudinal study. Developmental and behavioral characteristics of the ASD-M children compared to the ASD-N group were compared by using standardized scores.

**Results:**

20,8% of the children sample met criteria for macrocephaly. HC values became indicative of macrocephaly in the ASD-M group at the age range from 1 to 6 months, and persisted thereafter throughout the first five years of age. ASD-M children showed significantly higher developmental quotients of Griffiths III B and D subscales compared to ASD-N group. No significant differences in the severity of ASD symptoms assessed by ADOS-2 were observed between ASD-M and ASD-N groups.

**Conclusion:**

In this study HC size from birth to 5 years links to accelerated HC growth rate as early as the first 6 months of age in children with ASD and macrocephaly, preceding the onset and diagnosis of ASD. We found that in early childhood, children with ASD-M may exhibit some advantages in language and social communication and emotional skills without differences in autism severity, when compared with age-matched normocephalic ASD children. Longitudinal analyses are required to catch-up prospectively possible relationships between head size as proxy measure of brain development and neuro-developmental and behavioral features in children with ASD.

## 1 Introduction

Autism Spectrum Disorder (ASD) is a neurodevelopmental disorder characterized by social communication impairment and restricted, repetitive and stereotyped patterns of behaviors, interests or activities (1). The rising global prevalence rate of ASD (2) and the complexity of the ASD etiology, clinical features and developmental trajectories, have prompted intensive research to identify specific biological markers and endophenotypes for earlier diagnosis and treatment (3–5). Accelerated head growth associated with brain enlargement is a commonly reported biological feature of ASD, affecting 14%–34% of patients (4, 6–10). Head circumference (HC) is an accurate, rapid, and inexpensive tool used by research as a proxy measure of brain growth in the assessment of children with ASD (11).

Macrocephaly (or macrocrania) is clinically defined as an abnormally large head with an occipitofrontal circumference (OFC) greater than the 97th percentile.

A higher frequency of head overgrowth has been reported in children with ASD compared to typically developing children (TD) at varying age ranges, emphasizing a certain variability HC with respect to gender and age in ASD subjects (5). The abnormal head growth trajectory starts early in postnatal life and continues until at least 5 years of age (12). Early cerebral overgrowth in children with ASD may be followed by volumetric regression throughout the childhood (13). However, a recent longitudinal study showed the persistence of brain enlargement from early to late childhood in a subset of patients with ASD compared to age and sex-matched control subjects. This finding was correlated with a greater increase in white matter volume and a slower decrease in grey matter volume over time in ASD patients (14). Neuroimaging studies described a generalized enlargement of frontal, temporal and parietal lobes, involving both gray and white matter (15) or mainly limited to the frontal lobe gray matter (13, 16).

Altogether, previous findings have generally been interpreted as reflecting excessive neurogenesis/neuronal proliferation and inappropriate synaptic pruning, which may underlie the increased brain size in patient subsets with ASD (17, 18). These neuropathological abnormalities would also result in cortical surface area overgrowth (19), especially in cortical areas related to sensory information processing (middle occipital cortex) in children at high risk for ASD than in those at low or no risk for ASD. Thus over-expansion of cortical surface area and related head size increase may represent an early event in a cascade leading to brain overgrowth and emerging ASD symptoms (20), supporting identification of children at risk of ASD with or without a history of regression (8, 10, 21).

The timing of HC increase in children with ASD and its relationship to the appearance of behavioral symptoms is unclear so far. Overall, the results of studies are inconsistent regarding age, gender and intelligence quotient (IQ) effects on the HC growth rate and the relationship of macrocephaly to the clinical features of ASD and their severity (8, 22–24).

Clinical variables have been explored in order to identify meaningful subgroups that may share common genetic underpinning (8). Macrocephaly has been described as a clinical indicator of genetic subtypes of ASD. Historically, mutations in the gene phosphatase and tensin homolog (PTEN) were detected in a subset of individuals with large head and ASD (25).

Dysregulation of brain developmental processes due to multiple genomic variations in genes involved in cell proliferation (e.g., PTEN, mTOR pathway), chromatin remodeling (e.g., chromodomain helicase DNA binding protein 8, CHD8), protein transcription and translation and biological adhesion (WNT pathway) has been associated with ASD and macrocephaly (26–30), in order to provide elucidation of genotype–phenotype correlations and new insights into different subtypes of ASD. Approximately 17–20% of children with ASD and macrocephaly have pathogenic PTEN mutations (27) showing a distinct neurobehavioral phenotype with cognitive impairment extended to adaptive behavior, sensory deficits, repetitive behavior and decreased memory function (31–33).

In the effort to correlate different patterns of brain growth during development with the heterogeneous neurodevelopment trajectories of ASD in different subsets of affected individuals, the need for longitudinal analyses has been highlighted to carry out meaningful phenotyping (12, 22).

The present study was undertaken to assess longitudinal changes in head circumference by cross-sectional analyses in a sample of ASD children, with the aim of detecting significant differences in the growth trajectory of head circumference in children with macrocephaly compared to normocephalic children, in the first five years of life. We foresee that this study may identify a possible endophenotype of ASD associated with macrocephaly examining clinical correlates in the two groups of patients.

## 2 Materials and methods

### 2.1 Study design

This cross-sectional study was performed in two phases. In the first one, HC measurements were collected from birth to 5 years of age in a sample of children with ASD consecutively observed during the study period. In the second phase, we compared ASD children with macrocephaly (ASD-M) and normocephalic children (ASD-N) using neurodevelopmental and neurobehavioral assessment tools.

Data used for this study were collected from the clinical files and obtained as part of clinical protocols for patients with ASD. The study was approved by the local Ethics Committee of Catania University Hospital. All procedures performed in the present study were in accordance with the 1964 Declaration of Helsinki and its later amendments (2013). Written informed consent was obtained from both parents of each participant.

#### 2.1.1 Participants and data collection procedure

At study entry, 79 subjects were recruited, between October 2022 and July 2023, from a clinical population with ASD, diagnosed at the Child Neurology and Psychiatry Unit, Department of Clinical and Experimental Medicine, University of Catania. Longitudinal auxological data (HC, height and weight) were collected from birth to 5 years of age using medical records from the Child Neurology and Psychiatry Unit. Data on gestational age at birth were also collected.

The Inclusion criteria were as follows: 1) a clinical diagnosis of ASD according to DSM-5 criteria and all testing measures, including the Autism Diagnostic Interview-Revised (ADI-R) and the Autism Diagnostic Observation Schedule, 2nd edition (ADOS-2); 2) age at study time ≤ 5 years; 3) at least 3 HC measurements in the time period between 0 and 5 years of age.

Exclusion criteria included: 1) general overgrowth (head circumference, height and weight >97^th^ percentile/>2 standard deviations, SD); 2) presence of microcephaly (head circumference <3^rd^ percentile/<-2 SD); 3) lack of repeated measures of HC in the first five years of age.

All HC data refer to measurements obtained manually, using a non-stretchable tape measure placed over the maximum fronto-occipital head circumference.

HC, body length and weight measures at birth were converted into percentiles using the Italian Neonatal Study (INeS) charts, promoted by the Italian Society of Neonatology.

The Growth4 Software was used to calculate percentiles and SD values for each available measurement from the postnatal period to 5 years of age, according to the World Health Organization (WHO) growth charts.

HC measurements were normalized for sex and age by conversion to Z-scores based on the WHO mean values for healthy infants (World Health Organization 2006, Child Growth Standards).

Macrocephaly was defined as a HC greater than the 97^th^ percentile, that is more than 1.88 SD above the normative mean (z score > 1.88) (8, 24, 34).

Participants were classified into two groups: ASD macrocephaly (ASD-M, Z-scores ≥1.88 in at least two consecutive HC measurements), and ASD non-macrocephaly (ASD-N).

Based on the distribution of HC measurements (Z-scores) in the study sample, five age ranges were identified: birth, 1–6 months, 8–18 months, 20–32 months, 33–60 months. In each age range, ASD-M and ASD-N patients were matched for age and sex.

All participants underwent neuropsychiatric assessment and clinical data were compared between the two study groups ASD-M/ASD-N. The clinical diagnosis of ASD was confirmed using the Autism Diagnostic Observation Schedule, second edition (ADOS-2). The Griffiths Scales of Childhood Development - 3rd edition (Griffiths III) was administered in order to assess the psychomotor development.

### 2.2 Standardized measures

Symptoms of ASD were established using the gold-standard tools for ASD diagnosis: Autism Diagnostic Interview-Revised (ADI-R) (35) and the Autism Diagnostic Observation Schedule, 2nd edition (ADOS-2) (36). The ADOS-2 is a semi-structured, standardized assessment of core deficits in ASD. It contains five modules that are differentiated by children’s developmental and language levels. In the present study participants completed the Toddler Module (designed specifically for children 12–30 months old with limited language), the Module 1 (used for children aged from 31 months who do not consistently use phrase speech) or the Module 2 in a minority of children using phrase speech, but who were not verbally fluent. To allow comparisons among different modules, ADOS-2 scores (total score, Social Affect, SA, and Restricted and Repetitive Behaviour, RRB, scores) were converted to respective calibrated severity scores (CSS 1–10 indicating absence to severe autism) (37–39).

An overall measure of children’s psychomotor development was provided by the Griffiths III assessment across five subscales (40). Subscale A (Foundations of Learning) assesses the ability of learning; subscale B (Language and Communication) evaluates the development of both receptive and expressive language and social communication abilities; subscale C (Eye and Hand Coordination) assesses visual perception and fine motor skills; subscale D (Personal-Social-Emotional) evaluates child’s ability to adapt, personal autonomy and early social and emotional development through items measuring imitation, joint attention, emotional recognition and empathy; subscale E (Gross Motor domains) refers to the child’s early development of postural control, gross body coordination, balance and visual-spatial coordination. Subscale raw scores and general development raw scores are calculated to determine the Developmental Age, Scaled Score and Development Quotient, according to the norm tables.

### 2.3 Statistical analysis

In the first phase of the study, the one-way ANOVA statistical test was initially applied to compare ages (months) as means (M) and standard deviations (SD) in the five subgroups (age ranges). Analysis of variance (ANOVA) was then performed to find out possible significant differences on HC measurements (z-scores) between the two groups (ASD-M/ASD-N) in each age range.

In the second phase of the study, we used the independent samples t-test to compare the mean scores on each clinical assessment measure between the two groups (ASD-M/ASD-N).

The statistical significance level α was established at 0.05. All statistical tests were performed by using SPSS version 27 (SPSS, Inc., Chicago, IL, USA, IBM, Somers, NY, USA).

## 3 Results

### 3.1 Comparison of HC growth in children with ASD in the period from 0 to 5 years of age

Out of an initial sample of 79 subjects with ASD, thirty-four patients were excluded due to the unavailability of repeated HC measurement during the age 0–5 years. Forty-five children with a confirmed diagnosis of ASD (male to female ratio = 5:1; mean age: 4.4 ± 1.1) were included in two groups (ASD-M/ASD-N) based on HC measurements (Z-score). Ten children (20,8%) met criteria for macrocephaly (Z-scores ≥1.88). Routine laboratory analyses, extended metabolic screening and array-CGH analyses yielded normal results. Two patients were diagnosed with germline PTEN mutations (c.697C>T/p.Arg233 and c.62T>G/pPhe21Cys, respectively).

Demographic characteristics in ASD-M/ASD-N groups are reported in **Table 1**.

**Table 1:** Demographic characteristics in ASD-M/ASD-N groups and in the total sample.

Participants	ASD-M *(N = 10)*	ASD-N *(N = 35)*	Total Sample *(N = 45)*
**Years of age (mean ± SD)**	4.2 ± 0.8	4.5 ± 1.1	4.4 ± 1.1
**Males**	7 (70%)	31 (88.6%)	38 (84.4%)
**Females**	3 (30%)	4 (11.4%)	7 (15.6%)
**Pre-term birth**	0	3 (8.6%)	3 (6.7%)
**At-term birth**	10 (100%)	32 (91.4%)	42 (93.3%)

ASD-M, macrocephalic group; ASD-N, normocephalic group; N, number of participants; SD, standard deviation.

At each study interval, the mean age was not significantly different between the two groups ASD-M/ASD-N (birth: *F*(37)=2.28, *p*=0.14; 1–6 months: *F*(17)=2.74, *p*=0.12; 8–18 months: *F*(18)=0.92, *p* =0.35; 20–32 months: *F*(23)=2.82, *p* =0.11; 33–60 months: *F*(28)=0.16, *p*=0.69).

HC Z-score data in the ASD-M group compared to the ASD-N group at each age range are reported in **Table 2**. At birth HC measurements were in the normal range in both groups. However, mean Z-scores were significantly higher in the ASD-M group than in the ASD-N group (*F*=10.2, *p*=0.004).

**Table 2:** Head circumference z-scores data in ASD-M group compared to ASD-N group at each age interval.

Age (months)	Study groups	Z-score means (± SD)	*F*	*p*
**Birth**	ASD-M	0.9 (± 0.4)	10.2	0.004*
	ASD-N	-0.02 (± 0.7)		
**1–6**	ASD-M	1.6 (± 1.3)	8.7	0.009*
	ASD-N	0.2 (± 0.8)		
**8–18**	ASD-M	1.5 (± 0.8)	5.1	0.036*
	ASD-N	0.6 (± 0.8)		
**20–32**	ASD-M	2.1 (± 0.7)	21.3	<0.0001*
	ASD-N	0.4 (± 0.9)		
**33–60**	ASD-M	2.2 (± 0.3)	29.6	<0.0001*
	ASD-N	0.6 (± 0.7)		

ASD-M, macrocephalic group; ASD-N, normocephalic group; N, number of participants; SD, standard deviation.

*p<0.05, significant difference.

**Figure 1:**
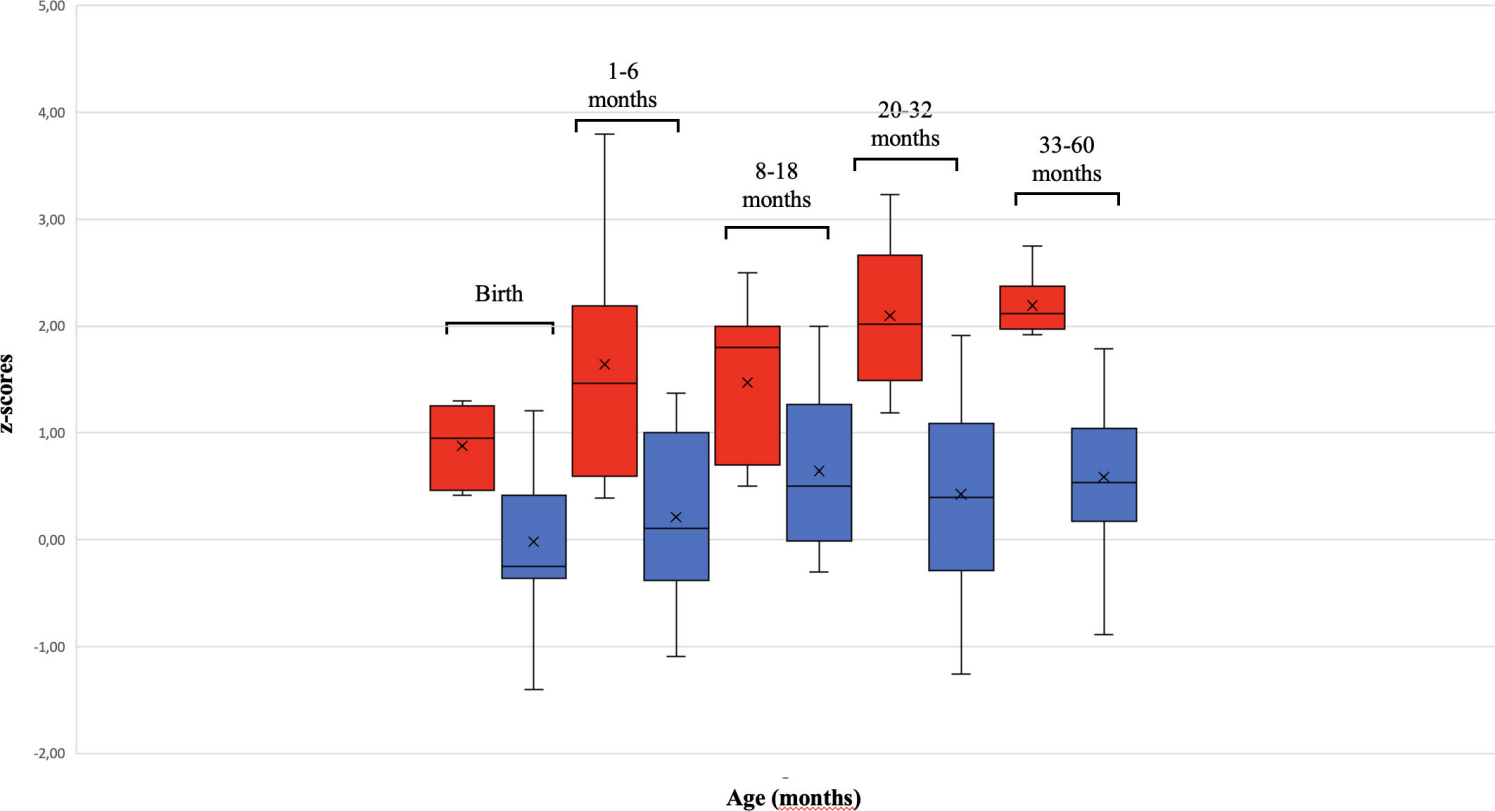
Differences in HC growth between ASD-M and ASD-N children from birth to 60 months. The box plot shows that children with Autism Spectrum Disorder and macrocephaly (ASD-M, red) had a more rapid HC growth than ASD normocephalic subjects (ASD-N, blue). The two patients harboring PTEN mutations (outliers) were not included in the HC growth analysis.

We found a significant increase of HC in the ASD-M group compared to the ASD-N group at each age interval (**Table 2**) suggesting that ASD-M patients showed an excessive HC growth in the study period than ASD-N subjects (**Figure 1**).

### 3.2 Neurobehavioral phenotype comparison between ASD-M and ASD-N children

In the second phase of the study, we sought to determine whether and to what extent the developmental and behavioral characteristics of the ASD-M group differed from the ASD-N group, by comparing the mean scores obtained on each clinical assessment tool (**Table 3**). At the time of assessment, chronological age (CA) (ASD-M mean age: 29.7 ± 9.4 months; ASD-N mean age: 34.6 ± 9.3 months) was not significantly different between the two groups (t=1.4, p=0.08), ruling out that possible clinical differences might be related to different CA in the group comparison.

**Table 3:** Comparison of the Griffiths III and ADOS-2 scores between ASD-M and ASD-N study groups.

Clinical assessment		Group	Mean (± SD)	*t*	*p*
Griffiths III	DQA	ASD-M	77.1 (± 22.5)	0.9	0.38
		ASD-N	70.6 (± 19)		
	DQB	ASD-M	65 (± 17.1)	3.4	0.0016*
		ASD-N	51.9 (± 8.1)		
	DQC	ASD-M	74.3 (± 20.2)	1.1	0.27
		ASD-N	67 (± 17)		
	DQD	ASD-M	70.4 (± 16.7)	2.2	0.032*
		ASD-N	58.4 (± 14.1)		
	DQE	ASD-M	84.6 (± 20.1)	1.1	0.28
		ASD-N	76.5 (± 19.7)		
ADOS-2	SA CSS	ASD-M	7.3 (± 2.3)	0.1	0.45
		ASD-N	7.2 (± 2.5)		
	RRB CSS	ASD-M	6.9 (± 1.9)	0.8	0.22
		ASD-N	6.2 (± 2.7)		
	CSS TOT	ASD-M	7.2 (± 2.4)	1.1	1.14
		ASD-N	7.0 (± 2.3)		

ASD-M, macrocephalic group; ASD-N, normocephalic group; SD, standard deviation; DQA, Subscale A Development Quotient; DQB, subscale B Development Quotient; DQC, subscale C Development Quotient; DQD, subscale D Development Quotient. DQE, subscale E Development Quotient; ADOS-2, Autism Diagnostic Observation Schedule; SA CSS, Social Affect Calibrated Severity Score; RRB CSS, Repetitive and Restrictive Behavior Calibrated Severity Scores; CSS TOT, Calibrated Severity Total Score.

We first investigated about differences in the severity of autism symptoms by using the ADOS-2 total CSS. No significant differences were found comparing ASD-M children with ASD-N children (SA CSS: *t*=0.3, *p*=0.76; RRB CSS: *t*=0.8, *p*=0.81; CSS TOT: *t*=0.2, *p*=0.85).

As to the developmental profile on the Griffith III scales, the General Development Quotient (GDQ) was below the normal range in both groups, but was significantly higher in ASD-M children (mean GDQ = 68.2± 18.11) than in the ASD-N group (mean GDQ = 57.03 ± 12.77) (*t*= 2.16, *p*=0.02).

Then, development quotients (DQ) on each subscale of the Griffiths III were compared between the ASD-M and ASD-N groups. No significant differences were found for the subscale A (*t*=0.9, *p*=0.38), subscale C (*t*=1.1, *p*=0.27) and subscale E DQ (*t*=1.1, *p*=0.28). ASD-M children scored significantly higher on the subscale B (*t*=3.4, *p*=0.0016) and subscale D (*t*=2.2, *p*=0.032) than ASD-N children (**Table 3**).

## 4 Discussion

The present study aimed to investigate the growth trajectory of HC in a sample of ASD children in the first five years of life. We sought to determine whether macrocephaly was correlated with other clinical characteristics in ASD children profiling a possible endophenotype associated with macrocephaly.

Macrocephaly was consistently reported in children with ASD than in neurotypical peers (5, 8, 10, 41), with an overall prevalence rate of 15.7% vs 3%, respectively (4).

In this study 20.8% of the total sample of ASD children developed macrocephaly during the first 5 years of life, a finding consistent with rates reported in the ASD literature (11, 34, 42, 43).

We found that ASD patients with macrocephaly (ASD-M group) presented a significantly larger HC size at birth compared to normocephalic ASD children (ASD-N group), although birth Z-scores were in the normal range in both groups. This result might indicate a possible prenatal brain overgrowth, consistent with observed late-gestational fetal HC overgrowth among children with ASD (44, 45). Most studies reported normal HC at birth in ASD infants (6, 10, 12, 46–48), while a few studies described smaller HC at birth with an overt increase in later months in ASD infants compared to neurotypical children (11, 23).

In the current ASD-M sample, head circumference was found to be consistent with the definition of macrocephaly from the first six months of life (F=7.7, p=0.014) and persisted thereafter throughout the first five years of age. In line with previous studies, we found that an accelerated HC growth rate is present in the first year of life and precedes the onset and diagnosis in children with autism spectrum disorder (8, 10, 20, 21, 23). Increased rates of head growth in early childhood (13) were mantained until the age of 5 years in children with ASD (12, 46, this study) and were not followed by volumetric regression until at least late childhood (age 11) (14).

Brain size is positively correlated with cognitive function in typically developing individuals, however previous data are inconsistent regarding neurocognitive development in ASD children with macrocephaly (22). Moreover, brain and behavior relationships may develop at different times during development, illustrating the need of longitudinal analyses to achieve meaningful phenotyping. Notably, we found significant differences of ASD-M children compared to the ASD-N group, assessed by the Griffiths III developmental scales.

Increasing evidence suggests the validity of Griffiths III in describing specific developmental profiles in children with ASD. Recently different developmental profiles on the Griffiths III have been detected in children with ASD with respect to children with developmental delay (DD) (49). Griffiths III B and D-subscales, which probe language, social and emotional skills, have been shown to be the most impaired in ASD and the most predictive for ASD risk (49–53). In this regard, we recently developed a novel level-2 ASD screener, the Developmental Autism Early Screening (DAES), by selecting the most predictive Griffiths III B- and D-subscales items for ASD risk in the first three years of age, which may differentiate children with ASD-risk from their peers with DD or with typical development (TD) (53).

In the present study we found that the Griffiths III DQ on the B and D subscale were significantly higher in ASD-M than in ASD-N group. This indicates that at the study time, children with ASD and macrocephaly showed less impairment in language, communication, social and emotional skills compared with age-matched normocephalic ASD children.

An advantage in language development and general IQ measures was reported in ASD patients with macrocephaly (9). Abnormal acceleration of HC in early life was associated with better adaptive functioning and less impairment in social and behavioral domains in macrocephalic children compared to normocephalic children with ASD. This observation led to the hypothesis that the accelerated head growth in early childhood may be a protective reaction in response to pathognomonic neurodevelopmental processes that contribute to ASD (8).

On the other hand, additional studies yielded conflicting results or failed to detect significant differences in the DQ and IQ scores between macrocephalic and normocephalic ASD patients (12, 34, 43). Lower IQ scores might be associated with a history of language and social skills regression in ASD children with macrocephaly (10, 22, 54). In some instances, better non-verbal than verbal performances were correlated with increased head size in ASD patients (41).

These discrepancies in the clinical correlates of HC in ASD may reflect abnormalities in neurodevelopmental trajectories of ASD children whereby different skill domains become increasingly uneven over time (41).

The association between the peculiar neurodevelopmental profile of ASD-M children and the underlying neuroanatomical abnormalities and pathophysiological mechanisms needs to be further investigated. The degree, rate and/or duration of the brain overgrowth may be related to neuroanatomical and clinical outcome of ASD neurophenotypes. An inappropriate synaptic pruning or arborization with increased axon and dendrite number and size produces too many connections in various brain areas (18). The better performance of ASD-M children in language, social and emotional skills might suggest that the brain areas involved in these domains are not directly affected by abnormal growth processes or, alternatively, are more active due to compensatory mechanisms at certain times of neurodevelopment.

Otherwise, we found no significant differences between ASD-M and ASD-N groups in the severity of ASD symptoms assessed by ADOS-2. This result is consistent with other studies that did not identify differences on ADOS (10, 12, 22), ADI-R (24) and CARS (55), used to measure ASD severity in the studied groups of ASD patients with macrocephaly.

There are certain limitations in this study. The sample is relatively small and available measurements of head circumference are not uniform in all considered age ranges. Moreover, we focused on the first 5 years of life. Further investigations are needed to compare the two groups (ASD-M/ASD-N) at older ages, in order to detect differences in head growth and developmental trajectories. A few studies have suggested that the differences between ASD children with macrocephaly and ASD normocephalic children may persist at older ages (7, 56). It is not clear whether a brain size normalization occurs in adolescence: discrepancies between studies might be explained by the age heterogeneity of participants and a selection bias due to the inclusion of more compliant patients with higher IQs in neuroimaging studies (14). Imaging and EEG studies may be informative to further investigating additional features of the ASD-M endophenotype related to the neuro-behavioral profile. The co-occurrence of temporal EEG abnormalities, regression and macrocephaly has been described in a previous study, in order to define anatomic or pathophysiologic subtypes of ASD, highlighting the crucial role of the temporal region in processing language and social stimuli (57).

## 5 Conclusions

Alterations in brain organization and developmental trajectory might be strong biological indicators of ASD subtypes that would be associated with different patterns of behavioral symptoms or co-occurring conditions (22). In this study HC measurements from birth to 5 years links to early accelerated HC growth rate as early as the first 6 months of age in children with ASD and macrocephaly, preceding the onset and diagnosis of ASD. This observation concurs with recent findings illustrating that sub-regional brain fetal measurements at 20 weeks and fetal HC at 28 weeks were positively associated with Q-CHAT scores at 18–20 months of age (45). In addition, we demonstrate that in early childhood, children with ASD-M may exhibit some advantages in language and social communication and emotional skills without differences in autism severity, when compared with age-matched normocephalic ASD children. In this regard, we emphasize the need of longitudinal analyses to catch-up prospectively possible relationships between head size as proxy measure of brain development and neuro-developmental and behavioral features. Prospective investigations in larger samples may well consider including *ad-hoc* genetic and technical investigations to understand the precise nature of the association between accelerated head growth in ASD and the clinical phenotype. This future perspective may increase knowledge about clinical outcome and guide the therapeutic choices.

## Data availability statement

The original contributions presented in the study are included in the article/supplementary material. Further inquiries can be directed to the corresponding author.

## Ethics statement

The studies involving humans were approved by Comitato Etico Locale Catania 1. The studies were conducted in accordance with the local legislation and institutional requirements. Written informed consent for participation in this study was provided by the participants’ legal guardians/next of kin. Written informed consent was obtained from the minor(s)’ legal guardian/next of kin for the publication of any potentially identifiable images or data included in this article.

## Author contributions

LaC: Conceptualization, Data curation, Formal analysis, Methodology, Writing – original draft, Writing – review & editing. LuC: Conceptualization, Data curation, Methodology, Writing – original draft. LR: Conceptualization, Data curation, Methodology, Writing – original draft. AP: Writing – review & editing. MC: Methodology, Writing – review & editing. RR: Supervision, Writing – review & editing. RB: Conceptualization, Data curation, Formal analysis, Investigation, Methodology, Supervision, Writing – original draft, Writing – review & editing.
